# Bacterial community composition is an important predictor of surface soil fertility across different land use types: a case study in the Three Gorges Reservoir area

**DOI:** 10.7717/peerj.18959

**Published:** 2025-03-27

**Authors:** Lin Xu, Dandan Cheng, Liang Feng, Xuetian Lu, Sarah Ruffell, Hongmei Wang

**Affiliations:** 1 School of Environmental Studies, China University of Geosciences (Wuhan), Wuhan, China; 2 State Key Laboratory of Biogeology and Environmental Geology, China University of Geosciences (Wuhan), Wuhan, China; 3 Department of Biology, University of Waterloo, Waterloo, Canada

**Keywords:** Human disturbance, Temporal variation of bacterial community, 16S rRNA gene sequencing, Biolog ECO-plate

## Abstract

**Background:**

Surface soil is a vital component of terrestrial ecosystems and is of great importance for primary productivity. In Zhangjiachong, a small watershed in Zigui County, central China, human activity and erosion cause extensive surface soil degradation. It is still unclear as to what extent human activity influences soil fertility and soil microorganisms in this area.

**Methods:**

Soil samples were collected, during spring and autumn, across a series of land use types with different levels of human activity. We assessed soil fertility and microbial communities using 16S rRNA gene sequencing and Biolog ECO-plates.

**Results:**

The results showed that higher levels of human activity were associated with lower soil fertility and microbial metabolic activity, in addition to higher bacterial diversity. Moreover, human activity had negative effects on the relative abundances of Proteobacteria and Acidobacteriota, which were the key drivers of surface soil fertility. Conversely, stronger human activity was associated with lower abundance of Actinobacteriota. This study suggested that human activity had a negative influence on surface soil fertility, and bacterial community composition could be a good predictor of surface soil fertility.

## Introduction

The uppermost layer of soil, also known as the surface soil or topsoil, plays a critical role in sustaining life on Earth. For example, the topsoil provides ecosystem services relating to growing crops, raising livestock, as well as regulating of water and climates. The topsoil also play a key role in nutrient cycling, which sustain diverse plant, animal and microbial species, and are essential for ecosystem resilience and stability (Anikwe & Ife, 2023; Telo da Gama, 2023).

Soil microorganisms, particularly bacteria and fungi, are fundamental to the functioning of soil ecosystems, *e.g.*, maintaining soil health, fertility, and overall environmental quality (Xu et al., 2021a; Xu et al., 2021c). For instance, soil microbial activity can suppress plant pathogens through competition for nutrients and space, as well as by producing antibiotics that inhibit harmful organisms (Osburn et al., 2023). The microbial activity contributes to the formation of soil aggregates through the production of polysaccharides and other binding agents, the improvement of soil structure, the enhancement of water infiltration, and the increase of the soil’s ability to retain moisture (Saccá et al., 2017). In addition, soil microbes are pivotal in the recycling of nutrients such as carbon (C), nitrogen (N), phosphorus (P), sulfur (S) and potassium (K). They decompose organic matter, breaking it down into simpler compounds that plants can absorb, providing important ecosystem services (Saccá et al., 2017).

One key example of human activity on ecosystems is land use change. For instance, agricultural activities converse forests, grasslands and wetland into arable land, leading to habitat and biodiversity loss (Ros et al., 2004; Wang et al., 2001; Bucała, 2014; Zhang et al., 2016a). As topsoil erodes or degrades, the nutrient-rich layer that supports plant growth diminishes. This results in lower agricultural productivity and increased reliance on chemical fertilizers, which can further degrade soil health over time (Joergensen & Emmerling, 2006). In addition, trampling decreases vegetation cover, plant species, soil respiration rate and enzymatic activities (Ros et al., 2004). Land use changes affect soil nutrients, *e.g.*, transition of forests and shrub lands into croplands significantly decreased soil organic matter (SOM), total nitrogen (TN), total phosphorus (TP), available nitrogen (AN), and available phosphorus (AP) contents (Wang et al., 2001). The application of intercropping in cultivated lands is reported to create differences in microorganisms (He et al., 2013; Zhao et al., 2014). Human induced changes lead to a decline in microbial diversity and abundance, disrupting the essential processes of nutrient cycling (Huang et al., 2020). Previous studies examining the influence of human activity (or land use types) on soil attributes typically focus on a single (or several) soil fertility properties, *e.g.*, SOC or available N, P, K (Fusaro et al., 2019; Dror, Yaron & Berkowitz, 2022), making it difficult to make an overall prediction of soil fertility. Additionally, an integrated concept, ecosystem multifunctionality, has been proposed to describe the comprehensive traits of an ecosystem (Hector & Bagchi, 2007; Maestre et al., 2012; Manning et al., 2018; Sanderson et al., 2004). Exploring the effects of human activity on soil fertility in this integrated way has been shown to be a powerful tool in recent studies (Delgado-Baquerizo et al., 2017). However, our current understandings about the influence of human activity on surface soil fertility and microbial communities are still not clear.

The Three Gorges Reservoir area (111°34′E, 34°82′N), located in the upper reaches of the Yangtze River in China, is one of the most important ecological and agricultural bases in China. Due to the significant changes of land uses brought about by the Three Gorges Dam project, this region serves as a representative case for understanding the complex interactions between soil fertility, soil chemistry, soil microbiome, and human interventions (Meng, Fu & Yang, 2010; Iqbal et al., 2010; Lin et al., 2013; Yin et al., 2024). In this study, soils from land use with different intensity of human disturbance, namely, sloping crop lands (high human disturbance, see experimental design section), flat lands (medium human disturbance) and secondary forest (low human disturbance) were collected in a small watershed in the Three Gorges Reservoir area. We intended to explore how surface soil fertility varies among three types of land use and to identify the key factors mediating soil fertility in this area. Furthermore, we sampled the soil in spring and autumn to explore the temporal variation in soil fertility and microbial community. We hypothesized that (i) soils in areas with intensive human activity would exhibit lower surface soil fertility and microbial diversity, as human activities may disrupt nutrient accumulation and suppress microbial species diversity; (ii) soil fertility and microbial community composition would vary seasonally between spring and autumn; and (iii) microbial taxa with greater stress tolerance would dominate in high human activity habitats (*e.g.*, sloping lands), while those favoring nutrient-rich conditions would be more abundant in areas with lower human activity (*e.g.*, forests).

## Materials and Methods

### Experimental design and soil sampling

The study site was located at Zhangjiachong watershed (110°57′E, 30°46′N;133 to 632 m above sea level, 1.62 km^2^ area) which is part of the Three Gorges Reservoir area and about 12 km southwest of Three Gorges dam. According to the WorldClim database (Fick & Hijmans, 2017), annual mean temperature is 10 °C and annual precipitation is 1,200 mm in this area. The area is characterized by yellow-brown soils that originate from the weathering of granite and/or sandstone. According to China’s soil classification system, these soils are known as *Alfisols*, while in the United States, they are categorized as both *Alfisols* and *Ultisols* (Wang, Xu & Liu, 1988). On sloping terrains, typical landscape in this area, the granite-derived soils often have a sandy structure. The predominant land uses in this region include agricultural land (26.7%), forests (60.6%), orchards for economic crops (4.6%), grasslands (2.0%), and barren hillsides (4.9%). Soil erosion primarily happens on sloping croplands and in areas with sparse vegetation, especially hills with slopes over 25° (Meng et al., 2024).

Eleven sampling plots were selected according to land use types. The land use types were characterized by a gradient intensity of human activity (Table 1). We scored the human activity according to a previous study (Cheng et al., 2022), and whether one type of human activity existed or not in a given type of land use. We considered sloping lands to be the land use type influenced mostly by human activity (scored 6, Table 1), as the crop fields with *Brassica campestris* and *Arachis hypogaea* might be disturbed by agricultural activities, such as plowing, fertilizing, crop rotation, trampling, weeding, pest and disease controlling and harvesting. In addition, trampling, weeding, pests and disease controlling and harvesting were considered to exist in flat lands as well, because orange and tea pickings would occur there. We treated forests as the land use type with the lowest intensity of human activity, such as occasional logging.

**Table 1:** Empirical scoring of the strength of human activity on each land use type. Higher score implicates stronger human activity.

Land use types	Human activity types						Total score
	Plowing	Fertilizing	Crop rotation	Weeding	Pest and disease control	Harvest	
Sloping croplands	1	1	1	1	1	1	6
Flat croplands	0	1	0	1	1	1	4
Forests	0	0	0	0	0	1	1

To avoid sampling bias of plant attributes (as those plants are assumed to affect soil properties and hence soil microorganisms), a series of plant species were included within each type of land use. The three types of land use were: sloping croplands (sloping lands), flat croplands (flat lands), and secondary forests (forests). The predominate plants in sloping lands were orange (*Citrus reticulata*) and tea trees (*Camellia sinensis*), and crop species, *e.g.*, *B. campestris* (in spring) and *A. hypogaea* (in autumn). In total, six plots of sloping land were sampled, among which three were planted with hedgerows to prevent soil erosion (plot No. 1-6 in Table 2). The major plant species in flat lands included orange and tea trees, and there were no other trees nor crops. Two sampling plots were set up from flat land (plot No. 7, 8 in Table 2). We selected three kinds of forests, in which the constructive species were camphor tree (*Cinnamomum camphora*), camphor and tea trees, and *Fagus longipetiolata*. Three sampling plots were selected, one in each of the forest types (plot No. 9-11 in Table 2).

**Table 2:** Information of land use types, crop, and plant species in each plot.

Plot number	Land use type	Crops or plants	Hedgerows planted^a^
1	Sloping croplands	*Citrus reticulata*	Yes
2	Sloping croplands	*Camellia sinensis*	Yes
3	Sloping croplands	*Brassica campestris* (grown in spring) *Arachis hypogaea* (grown in autumn)	Yes
4	Sloping croplands	*Citrus reticulata*	No
5	Sloping croplands	*Camellia sinensis*	No
6	Sloping croplands	*Brassica campestris* (grown in spring) *Arachis hypogaea* (grown in autumn)	No
7	Flat croplands	*Camellia sinensis*	–
8	Flat croplands	*Citrus reticulata*	–
9	Forests	*Cinnamomum camphora*	–
10	Forests	*Cinnamomum camphora* and *Camellia sinensis*	–
11	Forests	*Fagus longipetiolata*	–

**Notes.**

a The hedgerows are not available in croplands and forests.

Soil samples were collected in spring (April) and autumn (September) 2016. The five-point sampling method was applied during soil collection (Han et al., 2024). Five soil cores were collected from each quadrat: one from each of the four corners and one from the center. These cores were then pooled together to form a composite sample. Due to the plot size and condition variation, the soil was sampled in three 2 m × 2 m quadrats for crop lands, and three 5 m × 5 m quadrats for forests. In the meantime, plant richness was recorded in each quadrat. To avoid high levels of plant residue in the shallow soil layer and to minimize the effect of soil depth on the microbial community, soil was sampled at a depth of 5–10 cm instead of the conventional 0–10 cm. In total, 66 soil samples were collected, with three replicates for each plot at each season. An overview of the plot information is available in Table 2. Photographs demonstrating the natural condition of each land use type sampled are in Fig. S1.

### Soil properties

Soil samples were air dried, and soil physicochemical parameters were measured as described previously (Xu et al., 2020; Xu et al., 2021c). Briefly, soil pH was determined by a pH meter (UB-7, Denver, USA) after 1 min of sonication and shaking for 30 min of soil solution (1:5 weight (soil)/volume (distilled water)). The air-dried moisture content (MC) was measured gravimetrically. Total N (TN), Total P and total potassium (TK) was determined by the semimicro-Kjeldahl methods (Bremner, 1996), Mo-Sb colorimetric method (Murphy & Riley, 1962) and flame atomic emission spectrophotometry, respectively. The available N in the soil was measured by the alkaline hydrolysis diffusion method (Mulvaney & Khan, 2001); available P by Olsen’s method (Olsen, Cole & Watanabe, 1954); available potassium (AK) was extracted with a NH_4_OAc leaching-flaming luminosity (Zhou, Zhang & Niklas, 2014), and soil organic carbon (SOC) was measured by the soil organic matter by K_2_Cr_2_O_7_- H_2_SO_4_ oxidation method (Nelson & Sommers, 1996).

### Assessment of soil fertility

To construct a soil fertility index, we selected seven key items: the SOC, total and available N, P, K contents. These screened items consisted of the key nutrient parameters which indicate the provision of nutrients for plant growth and microbial metabolism. The z-score approach (averaging the z-score values of the above-mentioned soil fertility parameters) was used to obtain a quantitative soil fertility index (Maestre et al., 2012). The data related to these soil environmental traits used in this study are offered in Data S1.

### Soil microbial Biolog incubation

The metabolic activity of the microbial community was measured using the Biolog ECO-plates (Biolog Inc., Hayward, CA, USA) (Garland & Mills, 1991). Soil samples were stored at 4 °C for 24 h prior to measurement. Before analysis, the samples were incubated at 25 °C for 12 h to stimulate microbial metabolic activity. Soil microorganisms were extracted with an aliquot of 0.5 g of soil sample mixed with 24.5 mL of saline solution (0.85% w/v). Then the solution was diluted 1000 times and moved into Biolog ECO-plates. The incubation was conducted in a light-free box (to stimulate the dark environment in soil) at 25 °C for 240 h (Manjunath et al., 2018). Average well color development (AWCD) was calculated to describe metabolic activity of the microbial community (Equation (1)).

(1) \begin{eqnarray*}AWCD=\sum (Ci-Ri)/31\end{eqnarray*}

where *Ci* is the optical density (at 590 nm) of each well with sample, while Ri is the average optical density of the control wells. Plates with an incubation time of 96 h were chosen for further analysis, because this time point represented the logarithmic phase of incubation, which could be a good proxy for microbial metabolic activity (Fig. S2).

### Bacterial 16S rRNA gene sequencing

Soil samples for sequencing analyses were stored at −20 °C in a refrigerator until measurement. Soil metagenomic DNA was extracted using the D5625-01 (Omega, Norcross, GA, USA) DNA extraction kit according to the manufacturer’s instruction. To ensure that contamination was avoided, we included negative H_2_O, no negative PCR mix and primer controls in the PCR reactions. For each sample, triplicate PCR reactions were performed. The V3-V4 hypervariable regions of the bacterial 16S rRNA gene were amplified using a PCR Amplifier (2720, ABI, USA), with the primer set 515F (5-GTGCCAGCMGCCGCGGTAA-3) and 806R (5-GGACTACVSGGGTATCTAAT-3) (Caporaso et al., 2011). The PCR reactions were performed in a 25 µL mixture and included 0.25 µL of Q5 high-fidelity DNA polymerase (M0491L; NEB, Ipswich, MA, USA), five µL of 5 × reaction buffer, five µL of 5 × GC buffer, five µL of dNTP (10 mM), one µL of forward primer (10 µM), one µL of reverse primer (10 µM), one µL of DNA template, and 11.25 µL of ddH_2_O. The thermal program for amplification was 5 min of initial denaturation at 98 °C, 10 s at 98 °C, 30 s at 50 °C, and 30 s at 72 °C for 30 cycles before a final extension of 5 min at 72 °C.

The amplicons with about 300 bp size were selected by running 2% agarose gel electrophoresis in 1.0× TAE buffer and were purified using the AxyPrep DNA Gel Extraction Kit (AP-GX-250; Axygen). After quantified on a microplate reader (FLX800; BioTek) with the Quant-iT PicoGreen dsDNA Assay Kit (P7589; Invitrogen, Waltham, MA, USA), the purified amplicons from one sample were pooled together and normalized in equimolar amounts.

DNA libraries were constructed with equimolar mixed amplicons (100 ng) according to the manufacturer’s instruction (Illumina). Finally, target DNA was sequenced on an Illumina MiSeq platform. The sequencing data is deposited in NCBI database. The accession number is PRJNA511782. The data also can be accessed *via* Figshare (https://doi.org/10.6084/m9.figshare.27367386).

### Bioinformatics

Paired-end reads were merged using FLASH software (Magoč & Salzberg, 2011). The QIIME2 pipeline was then employed for subsequent analyses (Bolyen et al., 2019). Quality controls were processed using “quality-filter q-score” with default parameters. The amplicon sequence variants (ASVs) were obtained using the DADA2 denoise algorithm (Callahan et al., 2016). Using the “qiime feature-table filter-features” command, ASVs were further filtered with the following criteria: ASVs present less than two times, less than two soil samples and those from mitochondria and chloroplasts. Using “qiime feature-table rarefy” command, all samples were rarefied to nine, 860 sequences per sample, based on the sample with the fewest reads (see details in the Qiime2 scripts in the Code S1). For taxonomic classification of the 16S rRNA gene, the SILVA ribosomal RNA gene database (Release 138) was employed (Quast et al., 2012). The original and rarefied datasets can be found at Data S2. The scripts and these datasets are also deposited at Figshare (https://doi.org/10.6084/m9.figshare.27367386).

### Statistical analysis

The normality of data distribution was assessed using the ‘shapiro.test’ function in R. To ensure comparability across different parameters, tests for differences in soil fertility, microbial metabolic activity, and bacterial diversity were conducted between seasons (Spring and Autumn) and across three land use types using consistent methods. As the Shannon diversity index did not meet the normality assumption, the non-parametric Kruskal-Wallis test was applied, utilizing the ‘kruskal’ function from the agricolae package v1.3-7 (De Mendiburu, 2023). Using the ‘vegan’ package v2.6.4 (Oksanen et al., 2011), non-metric multidimensional scaling (NMDS) and permutational multivariate analysis of variance (PERMANOVA) were used complementary to check the differences in bacterial β diversity between seasons and land use types. Random forest analysis was used to identify the biomarkers governing soil fertility, using the ‘randomForest’ package v4.7.1.1 (Liaw & Wiener, 2002). The significance of each biomarker’s importance with respect to the soil fertility index was assessed using the ‘rfPermute’ package v2.5.2 (Archer, 2013).

Evaluation of the direct and indirect effects of human activity, plant diversity, pH, and microorganisms on soil fertility was conducted by partial least squares path modeling (PLS-PM), using the ‘plspm’package v0.5.1 (Sanchez, 2013). In this model, the biomarkers (bacterial taxa) identified by random forest modeling were treated as the proxies of microbial composition, which was set as a latent variable, while the other factors were manifest variables. These latent variables included two or more manifest variables. The relative contribution of the manifest variables was estimated by their loadings in the model. The models were conducted for 999 permutations using bootstraps to obtain reliable estimates of path coefficients (representing the direction and strength of the linear relationships between variables and loadings of the indicators). All statistical analyses were conducted in R 4.3.3 (R Core Development Team, 2010). The R scripts used in this study can be accessed in the Code S2 or *via* Figshare (https://doi.org/10.6084/m9.figshare.27367386).

## Results

### Soil properties of different land use types

**Figure 1:**
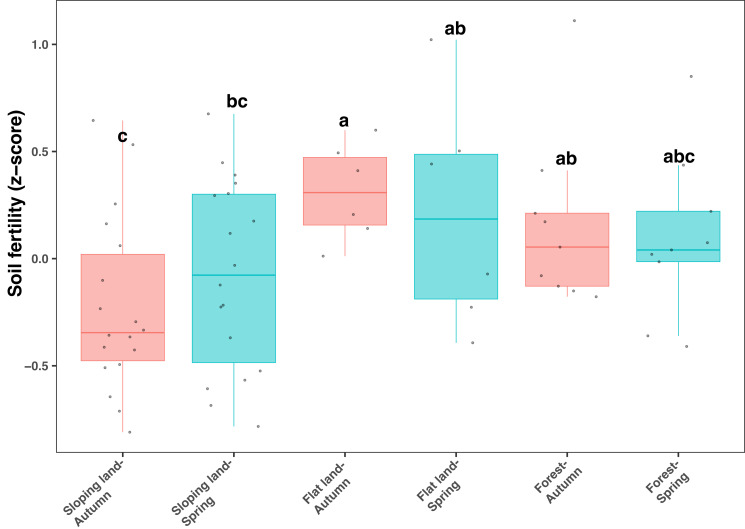
Box plots showing variation of soil fertility index among seasons and land use types. Different lowercase letters denote significant differences determined by the Kruskal-Wallis test at a significance level of *P* < 0.05.

All soil samples used in our study showed relatively low pH values (between five and six) and low organic C contents (from 10 to 20 mg kg^−1^, Table S1). Soil fertility index, calculated from total and available N, P, and K, and SOC, was significantly and positively correlated with soil organic C, total N, available N and available K (Fig. S3). In addition, SOC, AN and AK showed relatively higher correlation strength (Fig. S3). The soil fertility index significantly differed among different land use types (KW test: *χ*^2^=11.135, *df* = 5, *P* = 0.049), which was lower on sloping lands compared with other land use types (Fig. 1). However, the influence of season on soil fertility was not significant regarding each type of land use (Fig. 1). In addition, when land use was controlled, soil fertility was higher in orange plots (considering sloping lands only) and *Fagus* plots (considering forests only) than in plots with other plant species (Fig. S4).

### Variation of soil microbial communities

It is important to note that the rarefaction curve of each sample gradually reached a steady state with a sequencing depth higher than 5,000 sequences per sample (Fig. S5). Thus, resampling each sample to 9,860 sequences was enough to detect soil bacterial diversity. In total 29,294 ASVs were obtained; after filtration and resampling, the final dataset includes 12,683 ASVs (Data S2), which can be affiliated to 44 phyla 506 genera (Data S3–Data S4).

**Figure 2:**
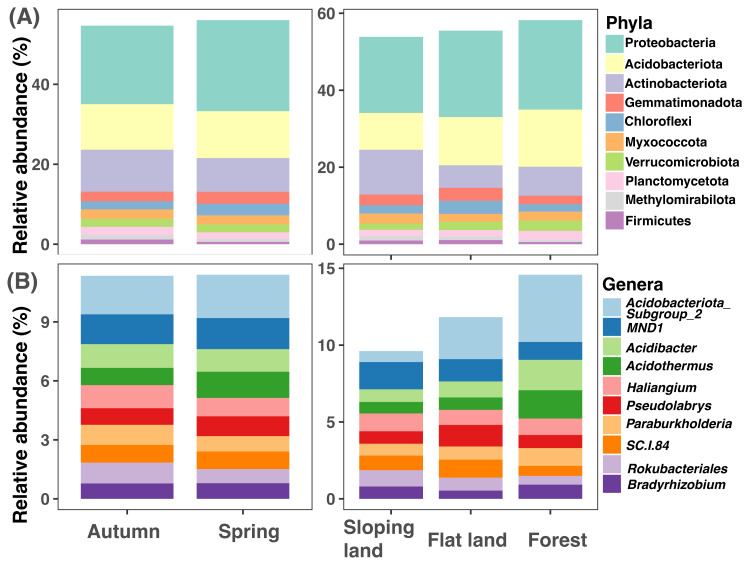
Variations of soil microbial community composition at phylum and genus levels across different seasons and land use types.

The top 10 soil bacterial phyla were Proteobacteria, Acidobacteriota, Actinobacteriota, Gemmatimonadota, Chloroflexi, Myxococcota, Verrucomicrobiota, Planctomycetota, Methylomirabilota, and Firmicutes (Fig. 2A). Proteobacteria was with higher amplicon relative abundances in samples collected in spring than those collected in autumn (paired Wilcox test: *W* = 256, *P* < 0.001); Proteobacteria (KW test: *χ*^2^=11.867, *df* = 2, *P* = 0.003) and Acidobacteriota (KW test: *χ*^2^= 45.134, *df* = 2, *P* < 0.001) had higher amplicon relative abundances in forests than croplands (sloping lands and flat lands). The highest amplicon relative abundance of Actinobacteriota was observed in sloping croplands (KW test: *χ*^2^= 39.94317, *df* = 2, *P* < 0.001, Fig. 2A).

The representative bacterial genera were *Subgroup2* of Acidobacteriota, *MND1, Acidibacter, Acidothermus, Haliangium, Pseudolabrys, Paraburkholderia, SC.I.84, Rokubacteriales, and Bradyrhizobium* (Fig. 2B). Amplicon relative abundance of *Haliangium* (paired Wilcox test: *W* = 201.5, *P* < 0.001) and *Rokubacteriales* (paired Wilcox test: *W* = 705, *P* = 0.040) were higher in autumn than spring; while *Subgroup2* (KW test: *χ*^2^= 43.854, *df* = 2, *P* < 0.001)*, Acidibacter* (KW test: *χ*^2^= 25.655, *df* = 2, *P* < 0.001) and *Acidothermus* (KW test: *χ*^2^= 21.769, *df* = 2, *P* < 0.001) were with greater dominance in forests than croplands (sloping lands and flat lands, Fig. 2B).

**Figure 3:**
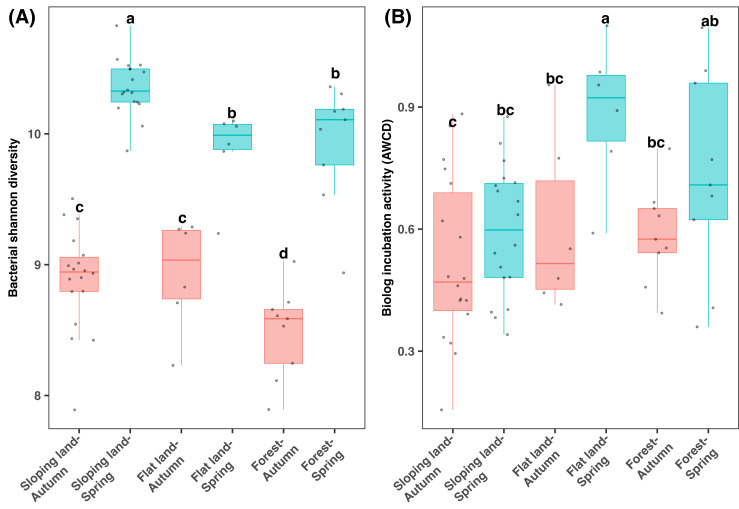
Box plots of bacterial diversity (A) and microbial metabolic activity (B) varying between seasons and land use types. Different lowercase letters denote significant differences determined by the Kruskal-Wallis test at a significance level of *P* < 0.05.

According to the 16S rRNA gene sequencing data, bacterial diversity was significantly differed among two seasons and land use types (KW test: *χ*^2^ = 51.832, *df* = 5, *P* < 0.001). The bacterial Shannon diversity was significantly higher in spring than autumn, and in cropland than forests (Fig. 3A). Regarding the same season and land use types, bacterial Shannon diversity showed minor difference among different plants, which was high in tea than orange plots in spring samples from sloping land and was lower in the *Fagus* forest than other plants in spring (Fig. S6).

Notably, differences in AWCD (which indicates microbial metabolic activity) among seasons were dependent on specific land use types (KW test: *χ*^2^=13.622, *df* = 5, *P* < 0.05), which was statistically similar between two seasons regarding sloping land and was higher in spring than autumn regarding flat land and forests (Fig. 3B). Additionally, in spring, sloping land demonstrated lower AWCD value than flatland and forests. A comparison of the samples from the same land use type indicated that AWCD was higher in orange plots (among all sloping land plots) and Camphor trees (among all forest plots) but was lowest in *Fagus* plots (among all the forest plots Fig. S7). In addition, AWCD differed in plots with or without hedgerows in the sloping lands (Fig. S7A).

**Figure 4:**
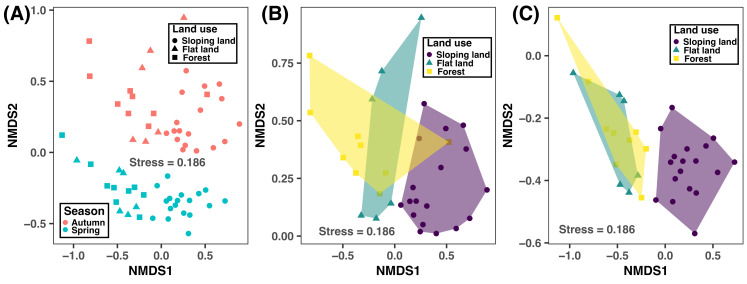
Non-metric multidimensional scaling (NMDS) plot showing the variation of bacterial community compositions at ASV level between different seasons and land use types based on the Bray–Curtis distance considering both seasons (A) and in autumn (B) and spring. Significance test results are provided in Table 3.

According to the NMDS plot and PERMANOVA results, bacterial community composition was significantly different between autumn and spring and across different land use types (Fig. 4 and Table 3). This suggested that human-induced changes in land use significantly altered soil bacterial compositions, regardless of sampling season considered.

### Environmental factors influencing soil microbial communities and soil fertility

**Figure 5:**
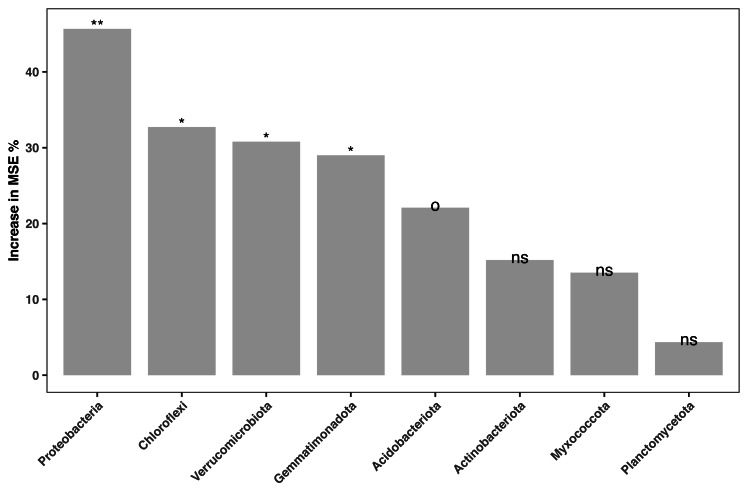
Bacterial biomarkers (at phylum level) of soil fertility identified by the random forest machine learning algorithm. An increase in the mean squared error (MSE%) indicates the importance to the accuracy of the model. The model’s explanatory power is 23.27%. Significance level: *ns*, not significant; *o*, *P* < 0.1; *, *P* < 0.05; **, *P* < 0.01; ***, *P* < 0.001.

The relative abundances of the 16S amplicon sequences related to key bacterial phyla were regressed against the soil fertility index in our random forest model. Only phyla with amplicon relative abundances higher than 0.01% were considered. The model accounted for 23.27% of the variance in soil fertility. Amplicon relative abundance of various phyla, such as Proteobacteria, Chloroflexi, Verrucomicrobiota and Gemmatinonadota and Acidobacteriaota were marginal (*P* < 0.1) or significant (*P* < 0.05) indicators of soil fertility in the study area (Fig. 5).

**Table 3:** Results of permutational multivariate analysis of variance analysis showing differences in community composition of soil bacteria between different seasons and land use types.

	Degree of freedom	Sum of squares	R2	F-value	*P*-value
Season	1	0.639	0.033	2.342	0.002
Land use	2	1.485	0.077	2.721	0.001
Season:Land use	2	0.778	0.04	1.426	0.023
Residual	60	16.371	0.849		
Total	65	19.273	1		

**Figure 6:**
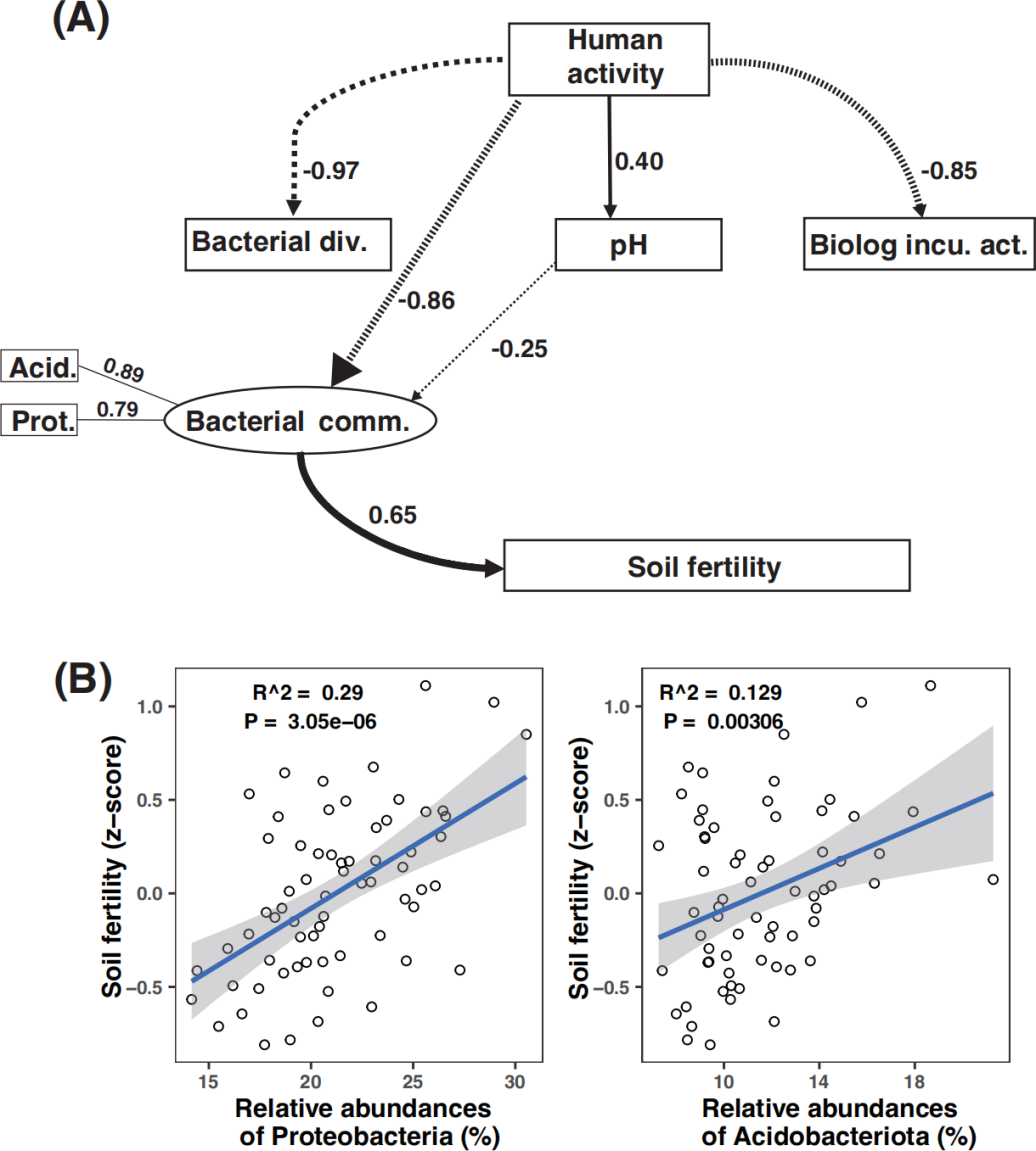
Partial least square path modeling (PLS-PM) showing the effects of human activity and biotic and abiotic factors on soil fertility (A) and associations between the major microbial attributes and soil fertility (B). The human activity in this model is scored according to the numbers of human activity relating to each land use type (Table 1). Arrows and path coefficients represent the direction and strength of the linear relationships between variables. Full lines indicate positive effects between blocks, and dotted lines indicate negative effects. Acid., Acidobacteriota; Bacterial comm., Bacterial community composition; Bacterial div., Bacterial diversity; Biolog incu. act., Biolog incubation activity; Prot., Proteobacteria; Plant diversity (the dominant plant species in the sample plot, see details in Table 2).

Path analysis demonstrated that bacterial community composition was the dominant driver of soil fertility. Moreover, microbial metabolic activity was directly and negatively affected by human activity. The negative effect of human activity on soil fertility was mitigated by the bacterial community composition (Fig. 6A). While amplicon relative abundances of Gemmatimonadota and Choroflexi were negatively correlated with soil fertility index (Fig. S8), the amplicon relative abundances of Acidobacteriota and Proteobacteria were the key drivers of soil fertility (Figs. S6B and S8).

## Discussion

### Land use types and above—ground plant species affect soil microbial communities

We recognize potential biases introduced during the amplification process that could affect the accuracy of taxonomic predictions. Nonetheless, we highlight that despite these limitations, our study provides valuable insights into the impact of micro-habitat factors on soil prokaryotic taxa. This is supported by numerous prior studies that have successfully used amplicon sequencing techniques to gather detailed community information on soil bacteria (Zhang et al., 2016b; Xu et al., 2020; Xu et al., 2021a; Xu et al., 2021b; Xu et al., 2024). Considering the disadvantages of Biolog ECO-plates and 16S rRNA gene sequencing, we combined both techniques to explore the soil microbial community. Previous studies suggested that Biolog ECO plate-based microbial metabolic activity in soils varied with land use (Rutgers et al., 2016), dominant vegetation (*C. camphor*, *Pinus massoniana*, or *Lespedeza bicolor)*, and root proximity (Tam et al., 2001). This study further demonstrated that both microbial metabolic activity and bacterial diversity varied among seasons and land use types. However, the patterns of microbial metabolic activity and bacterial diversity with respect to land use type differed (Fig. 2). These differences may be a consequence of the method used: Biolog ECO-plates and 16S rRNA gene sequencing. Firstly, Biolog ECO-plates are limited to measuring the microorganisms (*e.g.*, bacteria and fungi) which could utilize the 31 types of carbon sources, while 16S rRNA gene sequencing identifies bacteria, regardless of the carbon source used. Secondly, the Biolog ECO-plate incubation conditions favor fast growing microorganisms. Consequently, ECO-plates can identify microorganisms present in soil in small quantities or even in spore form, which become active after obtaining an easy carbon source. These organisms may not be identifiable using sequencing alone, due to low DNA concentrations in the soil sample. For example, the sloping croplands harbored a more diverse bacterial community than the forests, but most of these bacteria were in low amplicon relative abundance and did not survive Biolog incubation. The higher nutrient content and amplicon relative abundances of core bacterial taxa (*e.g.*, Acidobacteria and Proteobacteria) in forests, compared to sloping croplands, may lead to higher microbial metabolic activity and reduced bacterial diversity.

Within this watershed, human activity has led to distinct land use types. With the same soil type, different land use types showed different soil nutrient availability, bacterial diversity, and microbial metabolic activity (Figs. 1–4). Such differences were even observed among plots with different plant species. This is not surprising, since soil characteristics (Dybzinski et al., 2008; Huang et al., 2019) and microbial community are influenced by plant species (Burns et al., 2015), vegetation types, plant diversity and plant growth stages (Chen et al., 2013; Lozano et al., 2014; Phazna Devi et al., 2020; Zheng et al., 2021a; Zheng et al., 2021b; Zhu et al., 2021). In addition, we observed significant differences in soil fertility, microbial metabolic activity between soil with or without hedgerows in the sloping lands (Figs. S3–S5), highlighting the essential role of aboveground plants in controlling soil fertility and soil microbial communities. Our results suggested that activity and diversity of soil microorganisms varied among seasons and land use types. This further reflected the complex connections between land use, soil fertility, plant attributes, and soil microbial community.

The higher vegetation coverage in forests may leave more plant-derived organic matter in soils. Plant litter and root exudates may change the soil environment (Cotrufo et al., 2015). In addition, the decomposition of fallen oranges may also provide a driving force for higher soil organic matter. The accumulated organic matter may in turn retain moisture through the complex metabolic pathways of soil bacteria and therefore sustain higher soil fertility compared with other sampling plots. Moreover, the organic matter provided by root-associated processes could offer more substrates for microbial metabolism; consequently, the microorganisms selected by the Biolog ECO-plates were able to survive, because more than nine types of the carbon sources in the ECO-plates were consistent with root exudates (Choi & Dobbs, 1999). Therefore, it is reasonable that both high microbial metabolic activity and high soil fertility were observed in soils from forests and orange sloping lands.

### Human disturbances decrease surface soil fertility and alter soil microbial communities

Soil microbial communities are sensitive to disturbances (Shade et al., 2012). Sloping croplands underwent the greatest level of human activity, such as ploughing, rotation, fertilizing, and trampling. Disturbances in sloping croplands and crop rotation could introduce new microbial species into the soil and decrease the abundance of previously existing ones. Consequently, the number of microbial species in sloping croplands increased, while having an insignificant impact on soil function due to low abundance. Although previous studies have suggested that rare taxa can exhibit unique metabolic activities (Bickel & Or, 2021; Campbell et al., 2011) and are involved in particular metabolic functions, dominant species play a greater role in ecosystem functioning than subdominant and rare species (Treplin, Pennings & Zimmer, 2013; Xu et al., 2021b; Xu et al., 2021c). The flat croplands were agricultural lands that experienced trampling when oranges and tea were harvested, and the intensity of this type of disturbance was lower than that in sloping croplands. The forests should have the lowest level of disturbance caused by human activity. Thus, the introduction of non-native bacterial species would be lower in forest soils compared to flat croplands. Hence, soils in sloping croplands harbored more diverse bacterial species than flat croplands and forests, where the core amplicons were in higher relative abundances. These findings indicated that with a growing intensity of disturbance, soil microbial diversity and community composition might vary significantly.

The random forest and path modeling results showed that human activity had negative effects on soil fertility, and such negative effects were mitigated by the bacterial community composition. Similarly, the bacterial community composition was found to mitigate global change impacts (nitrogen addition, altered rainfall frequency) on ecosystem multifunctionality in a biocrust system (Liu et al., 2017). In addition, the results of path modeling signified that among all the factors we studied here, microbial community composition was the key factor mediating soil fertility. Analogously, the microbial community composition is also suggested to be the dominant factor controlling soil respiration rates in a cultivated land (Liu et al., 2018). Our study further indicated that the amplicon relative abundances of the core bacterial taxa (*i.e.,* Proteobacteria and Acidobacteriota) were important factors driving soil fertility, and these two phyla were more abundant in soils with higher fertility. Acidobacteriota are positively correlated with soil organic carbon (Vasileiadis et al., 2013). These taxa are well-known copiotrophs that thrive in nutrient-rich soil conditions (Yao et al., 2014; Yao et al., 2017; Xu et al., 2020). In addition, Proteobacteria are predicted to be copiotrophs (Goldfarb et al., 2011; Tong et al., 2021), which grow quickly and increase soil fertility through plant root-associated metabolic processes (Oh et al., 2012). The high amplicon relative abundance of Acidobacteria and Proteobacteria, along with their importance in mediating soil fertility, further supported the notions that abundant taxa are active and important in mediating biogeochemical cycling (Cottrell & Kirchman, 2003; Treplin, Pennings & Zimmer, 2013; Xu et al., 2021b; Xu et al., 2021c).

## Conclusion

Soil fertility is a good indicator of soil quality. Higher soil fertility may indicate a stronger ability of soil to support plant growth and microbial metabolism. This study demonstrated that surface soil fertility was lower in areas with higher levels of human activity and that microbial community composition was the key determinant of soil fertility under such conditions, giving new insights into the effects of human activity on soil attributes in this area. Contrary to bacterial diversity, soil fertility and microbial metabolic activity were higher in forests, which experienced lower human activity than other land use types. We also observed significant differences in the soil bacterial community composition at the temporal and land use scales. Higher levels of human activity decrease the amplicon relative abundances of Acidobacteriota and Proteobacteria, which were further identified to be the dominant taxa driving soil fertility. Further studies of community assembly processes and the potential interactions between bacterial species in our study area will provide significant insights into the microbial mechanisms maintaining soil fertility under different levels of human activity in the Three Gorges Reservoir area.

##  Supplemental Information

10.7717/peerj.18959/supp-1 Supplemental Information 1 Statistical summary of soil physicochemical properties in sampling plots with different land use types

10.7717/peerj.18959/supp-2 Supplemental Information 2 Supplemental Figures

10.7717/peerj.18959/supp-3 Supplemental Information 3 Qiime2 scripts used in this study

10.7717/peerj.18959/supp-4 Supplemental Information 4 R scripts used in this study

10.7717/peerj.18959/supp-5 Supplemental Information 5 Soil environmental trait of each samples used in this study

10.7717/peerj.18959/supp-6 Supplemental Information 6 Original and rarefied datasets of ASVs

10.7717/peerj.18959/supp-7 Supplemental Information 7 ASV abundunce in each sample (phyla level)

10.7717/peerj.18959/supp-8 Supplemental Information 8 ASV abundunce in each sample (genera level)

## Additional Information and Declarations

### DNA Deposition

The following information was supplied regarding the deposition of DNA sequences: The original sequencing reads are available at NCBI SRA: PRJNA511782) and at Figshare: Xu, Lin; Cheng, Dandan (2024). Bacterial community composition is an important predictor of surface soil fertility across different land use types: a case study in the Three Gorges Reservoir area. figshare. Dataset. https://doi.org/10.6084/m9.figshare.27367386.v1.

### Data Availability

The following information was supplied regarding data availability: Data: Soil physic - chemical properties of every soil samples (Data S1); The original and rarefied datasets of the ASVs (Data S2); amplicon numbers of ASVs affiliated to 44 phyla & 506 genera (Data S3 & Data S4). code: Qiime2 and R scripts used in this study.

## References

[ref-1] Anikwe MAN, Ife K (2023). The role of soil ecosystem services in the circular bioeconomy. Frontiers in Soil Science.

[ref-2] Archer E (2013). https://github.com/EricArcher/rfPermute.

[ref-3] Bickel S, Or D (2021). The chosen few-variations in common and rare soil bacteria across biomes. The ISME Journal.

[ref-4] Bolyen E, Rideout JR, Dillon MR, Bokulich NA, Abnet CC, Al-Ghalith GA, Alexander H, Alm EJ, Arumugam M, Asnicar F, Bai Y, Bisanz JE, Bittinger K, Brejnrod A, Brislawn CJ, Brown CT, Callahan BJ, Caraballo-Rodríguez AM, Chase J, Cope EK, Da Silva R, Diener C, Dorrestein PC, Douglas GM, Durall DM, Duvallet C, Edwardson CF, Ernst M, Estaki M, Fouquier J, Gauglitz JM, Gibbons SM, Gibson DL, Gonzalez A, Gorlick K, Guo J, Hillmann B, Holmes S, Holste H, Huttenhower C, Huttley GA, Janssen S, Jarmusch AK, Jiang L, Kaehler BD, Kang KB, Keefe CR, Keim P, Kelley ST, Knights D, Koester I, Kosciolek T, Kreps J, Langille MGI, Lee J, Ley R, Liu Y-X, Loftfield E, Lozupone C, Maher M, Marotz C, Martin BD, McDonald D, McIver LJ, Melnik AV, Metcalf JL, Morgan SC, Morton JT, Naimey AT, Navas-Molina JA, Nothias LF, Orchanian SB, Pearson T, Peoples SL, Petras D, Preuss ML, Pruesse E, Rasmussen LB, Rivers A, Robeson MS, Rosenthal P, Segata N, Shaffer M, Shiffer A, Sinha R, Song SJ, Spear JR, Swafford AD, Thompson LR, Torres PJ, Trinh P, Tripathi A, Turnbaugh PJ, Ul-Hasan S, van der Hooft JJJ, Vargas F, Vázquez-Baeza Y, Vogtmann E, Von Hippel M, Walters W, Wan Y, Wang M, Warren J, Weber KC, Williamson CHD, Willis AD, Xu ZZ, Zaneveld JR, Zhang Y, Zhu Q, Knight R, Caporaso JG (2019). Reproducible, interactive, scalable and extensible microbiome data science using QIIME 2. Nature Biotechnology.

[ref-5] Bremner JM, Sparks DL, Page AL, Helmke PA, Loeppert RH, Soltanpour PN, Tabatabai MA, Johnston CT, Sumner ME (1996). Nitrogen-total. Methods of soil analysis: part 3 chemical methods.

[ref-6] Bucała A (2014). The impact of human activities on land use and land cover changes and environmental processes in the Gorce Mountains (Western Polish Carpathians) in the past 50 years. Journal of Environmental Management.

[ref-7] Burns JH, Anacker BL, Strauss SY, Burke DJ (2015). Soil microbial community variation correlates most strongly with plant species identity, followed by soil chemistry, spatial location and plant genus. AoB Plants.

[ref-8] Callahan BJ, McMurdie PJ, Rosen MJ, Han AW, Johnson AJA, Holmes SP (2016). DADA2: high-resolution sample inference from Illumina amplicon data. Nature Methods.

[ref-9] Campbell BJ, Yu L, Heidelberg JF, Kirchman DL (2011). Activity of abundant and rare bacteria in a coastal ocean. Proceedings of the National Academy of Sciences of the United States of America.

[ref-10] Caporaso JG, Lauber CL, Walters WA, Berg-Lyons D, Lozupone CA, Turnbaugh PJ, Fierer N, Knight R (2011). Global patterns of 16S rRNA diversity at a depth of millions of sequences per sample. Proceedings of the National Academy of Sciences of the United States of America.

[ref-11] Chen F, Zheng H, Zhang K, Ouyang Z, Wu Y, Shi Q, Li H (2013). Non-linear impacts of *Eucalyptus* plantation stand age on soil microbial metabolic diversity. Journal of Soils and Sediments.

[ref-12] Cheng Y, Liu H, Chen D, Liu H (2022). Human activity intensity and its spatial–temporal evolution in China’s border areas. Land.

[ref-13] Choi KH, Dobbs FC (1999). Comparison of two kinds of Biolog microplates (GN and ECO) in their ability to distinguish among aquatic microbial communities. Journal of Microbiological Methods.

[ref-14] Cotrufo MF, Soong JL, Horton AJ, Campbell EE, Haddix ML, Wall DH, Parton WJ (2015). Formation of soil organic matter via biochemical and physical pathways of litter mass loss. Nature Geoscience.

[ref-15] Cottrell MT, Kirchman DL (2003). Contribution of major bacterial groups to bacterial biomass production (thymidine and leucine incorporation) in the Delaware estuary. Limnology and Oceanography.

[ref-16] De Mendiburu F (2023). https://CRAN.R-project.org/package=agricolae.

[ref-17] Delgado-Baquerizo M, Powell JR, Hamonts K, Reith F, Mele P, Brown MV, Dennis PG, Ferrari BC, Fitzgerald A, Young A, Singh BK, Bissett A (2017). Circular linkages between soil biodiversity, fertility and plant productivity are limited to topsoil at the continental scale. New Phytologist.

[ref-18] Dror I, Yaron B, Berkowitz B (2022). The human impact on all soil-forming factors during the Anthropocene. ACS Environmental Au.

[ref-19] Dybzinski R, Fargione JE, Zak DR, Fornara D, Tilman D (2008). Soil fertility increases with plant species diversity in a long-term biodiversity experiment. Oecologia.

[ref-20] Fick SE, Hijmans RJ (2017). WorldClim 2: new 1-km spatial resolution climate surfaces for global land areas. International Journal of Climatology.

[ref-21] Fusaro C, Sarria-Guzmán Y, Chávez-Romero YA, Luna-Guido M, Muñoz Arenas LC, Dendooven L, Estrada-Torres A, Navarro-Noya YE (2019). Land use is the main driver of soil organic carbon spatial distribution in a high mountain ecosystem. PeerJ.

[ref-22] Garland JL, Mills AL (1991). Classification and characterization of heterotrophic microbial communities on the basis of patterns of community-level sole-carbon-source utilization. Applied and Environmental Microbiology.

[ref-23] Goldfarb KC, Karaoz U, Hanson CA, Santee CA, Bradford MA, Treseder KK, Wallenstein MD, Brodie EL (2011). Differential growth responses of soil bacterial taxa to carbon substrates of varying chemical recalcitrance. Frontiers in Microbiology.

[ref-24] Han Z, Fu X, Yu J, Zhang H (2024). Detecting 3D salinity anomalies from soil sampling points: a case study of the Yellow River delta, China. Land.

[ref-25] He Y, Ding N, Shi J, Wu M, Liao H, Xu J (2013). Profiling of microbial PLFAs: implications for interspecific interactions due to intercropping which increase phosphorus uptake in phosphorus limited acidic soils. Soil Biology and Biochemistry.

[ref-26] Hector A, Bagchi R (2007). Biodiversity and ecosystem multifunctionality. Nature.

[ref-27] Huang L, Bai J, Wen X, Zhang G, Zhang C, Cui B, Liu X (2020). Microbial resistance and resilience in response to environmental changes under the higher intensity of human activities than global average level. Global Change Biology.

[ref-28] Huang X, Li S, Li S, Ye G, Lu L, Zhang L, Yang L, Qian X, Liu J (2019). The effects of biochar and dredged sediments on soil structure and fertility promote the growth, photosynthetic and rhizosphere microbial diversity of *Phragmites communis (Cav.) Trin. ex Steud*. Science of the Total Environment.

[ref-29] Iqbal J, Hu R, Feng M, Lin S, Malghani S, Ali IM (2010). Microbial biomass, and dissolved organic carbon and nitrogen strongly affect soil respiration in different land uses: a case study at Three Gorges Reservoir Area, South China. Agriculture, Ecosystems & Environment.

[ref-30] Joergensen RG, Emmerling C (2006). Methods for evaluating human impact on soil microorganisms based on their activity, biomass, and diversity in agricultural soils. Journal of Plant Nutrition and Soil Science.

[ref-31] Liaw A, Wiener M (2002). Classification and regression by random forest. R News.

[ref-32] Lin S, Iqbal J, Hu R, Shaaban M, Cai J, Chen X (2013). Nitrous oxide emissions from yellow brown soil as affected by incorporation of crop residues with different carbon-to-nitrogen ratios: a case study in Central China. Archives of Environmental Contamination and Toxicology.

[ref-33] Liu Y-R, Delgado-Baquerizo M, Trivedi P, He J-Z, Wang J-T, Singh BK (2017). Identity of biocrust species and microbial communities drive the response of soil multifunctionality to simulated global change. Soil Biology and Biochemistry.

[ref-34] Liu Y-R, Delgado-Baquerizo M, Wang J-T, Hu H-W, Yang Z, He J-Z (2018). New insights into the role of microbial community composition in driving soil respiration rates. Soil Biology and Biochemistry.

[ref-35] Lozano YM, Hortal S, Armas C, Pugnaire FI (2014). Interactions among soil, plants, and microorganisms drive secondary succession in a dry environment. Soil Biology and Biochemistry.

[ref-36] Maestre FT, Quero JL, Gotelli NJ, Escudero A, Ochoa V, Delgado-Baquerizo M, García-Gómez M, Bowker MA, Soliveres S, Escolar C, García-Palacios P, Berdugo M, Valencia E, Gozalo B, Gallardo A, Aguilera L, Arredondo T, Blones J, Boeken B, Bran D, Conceição AA, Cabrera O, Chaieb M, Derak M, Eldridge DJ, Espinosa CI, Florentino A, Gaitán J, Gatica MG, Ghiloufi W, Gómez-González S, Gutiérrez JR, Hernández RM, Huang X, Huber-Sannwald E, Jankju M, Miriti M, Monerris J, Mau RL, Morici E, Naseri K, Ospina A, Polo V, Prina A, Pucheta E, Ramírez-Collantes DA, Romão R, Tighe M, Torres-Díaz C, Val J, Veiga JP, Wang D, Zaady E (2012). Plant species richness and ecosystem multifunctionality in global drylands. Science.

[ref-37] Magoč T, Salzberg SL (2011). FLASH: fast length adjustment of short reads to improve genome assemblies. Bioinformatics.

[ref-38] Manjunath M, Kumar U, Yadava RB, Rai AB, Singh B (2018). Influence of organic and inorganic sources of nutrients on the functional diversity of microbial communities in the vegetable cropping system of the Indo-Gangetic plains. Comptes Rendus. Biologies.

[ref-39] Manning P, Van der Plas F, Soliveres S, Allan E, Maestre FT, Mace G, Whittingham MJ, Fischer M (2018). Redefining ecosystem multifunctionality. Nature Ecology & Evolution.

[ref-40] Meng QH, Fu BJ, Yang LZ (2010). Effects of land use on soil erosion and nutrient loss in the Three Gorges Reservoir Area, China. Soil Use & Management.

[ref-41] Meng X, Zhu Y, Shi R, Yin M, Liu D (2024). Rainfall—runoff process and sediment yield in response to different types of terraces and their characteristics: a case study of runoff plots in Zhangjiachong watershed, China. Land Degradation & Development.

[ref-42] Mulvaney RI, Khan SA (2001). Distinctive bacterial communities in the rhizoplane of four tropical tree species. Soil Science Society of America Journal.

[ref-43] Murphy J, Riley JP (1962). A modified single solution method for the determination of phosphate in natural waters. Analytica Chimica Acta.

[ref-44] Nelson DW, Sommers LE, Sparks DL, Page AL, Helmke PA, Loeppert RH, Soltanpour PN, Tabatabai MA, Johnston CT, Sumner ME (1996). Total carbon, organic carbon, and organic matter. Methods of soil analysis: part 3 chemical methods.

[ref-45] Oh YM, Kim M, Lee-Cruz L, Lai-Hoe A, Go R, Ainuddin N, Rahim RA, Shukor N, Adams JM (2012). Distinctive bacterial communities in the rhizoplane of four tropical tree species. Microbial Ecology.

[ref-46] Oksanen J, Blanchet FG, Kindt R, Legendre P, Minchin PR, O’hara R, Simpson GL, Solymos P, Stevens MHH, Wagner H (2011). R Package Version.

[ref-47] Olsen SR, Cole CV, Watanabe FS (1954). Estimation of available phosphorus in soils by extraction with sodium bicarbonate.

[ref-48] Osburn ED, Yang G, Rillig MC, Strickland MS (2023). Evaluating the role of bacterial diversity in supporting soil ecosystem functions under anthropogenic stress. ISME Communications.

[ref-49] Phazna Devi TA, Sahoo D, Setti A, Sharma C, Kalita MC, Indira Devi S (2020). Bacterial rhizosphere community profile at different growth stages of Umorok (*Capsicum chinense*) and its response to the root exudates. International Microbiology.

[ref-50] Quast C, Pruesse E, Yilmaz P, Gerken J, Schweer T, Yarza P, Peplies J, Glöckner FO (2012). The SILVA ribosomal RNA gene database project: improved data processing and web-based tools. Nucleic Acids Research.

[ref-51] R Core Development Team (2010). http://www.R-project.org.

[ref-52] Ros M, Garcia C, Hernandez T, Andres M, Barja A (2004). Short-term effects of human trampling on vegetation and soil microbial activity. Communications in Soil Science and Plant Analysis.

[ref-53] Rutgers M, Wouterse M, Drost SM, Breure AM, Mulder C, Stone D, Creamer RE, Winding A, Bloem J (2016). Monitoring soil bacteria with community-level physiological profiles using Biolog™ECO-plates in the Netherlands and Europe. Applied Soil Ecology.

[ref-54] Saccá ML, Barra Caracciolo A, Di Lenola M, Grenni P, Lukac M, Grenni P, Gamboni M (2017). Ecosystem services provided by soil microorganisms. Soil biological communities and ecosystem resilience.

[ref-55] Sanchez G (2013). PLS path modeling with R. Trowchez Editions.

[ref-56] Sanderson MA, Skinner RH, Barker DJ, Edwards GR, Tracy BF, Wedin DA (2004). Plant species diversity and management of temperate forage and grazing land ecosystems. Crop Science.

[ref-57] Shade A, Peter H, Allison SD, Baho DL, Berga M, Bürgmann H, Huber DH, Langenheder S, Lennon JT, Martiny JBH, Matulich KL, Schmidt TM, Handelsman J (2012). Fundamentals of microbial community resistance and resilience. Frontiers in Microbiology.

[ref-58] Tam L, Derry AM, Kevan PG, Trevors JT (2001). Functional diversity and community structure of microorganisms in rhizosphere and non-rhizosphere Canadian arctic soils. Biodiversity and Conservation.

[ref-59] Telo da Gama J (2023). The role of soils in sustainability, climate change, and ecosystem services: challenges and opportunities. Ecologies.

[ref-60] Tong D, Li Z, Xiao H, Nie X, Liu C, Zhou M (2021). How do soil microbes exert impact on soil respiration and its temperature sensitivity?. Environmental Microbiology.

[ref-61] Treplin M, Pennings SC, Zimmer M (2013). Decomposition of leaf litter in a U.S. saltmarsh is driven by dominant species, not species complementarity. Wetlands.

[ref-62] Vasileiadis S, Puglisi E, Arena M, Cappa F, Van Veen JA, Cocconcelli PS, Trevisan M (2013). Soil microbial diversity patterns of a lowland spring environment. FEMS Microbiology Ecology.

[ref-63] Wang J, Fu B, Qiu Y, Chen L (2001). Soil nutrients in relation to land use and landscape position in the semi-arid small catchment on the loess plateau in China. Journal of Arid Environments.

[ref-64] Wang Q, Xu F, Liu Z (1988). Tentative research on genetic characteristics of zonal soils in Hubei Province. Journal of Huazhong Agricultural University.

[ref-65] Xu L, He N, Li X, Cao H, Li C, Wang R, Wang C, Yao M, Zhou S, Wang J (2021a). Local community assembly processes shape β-diversity of soil phoD-harbouring communities in the Northern Hemisphere steppes. Global Ecology and Biogeography.

[ref-66] Xu L, Li X, Tang X, Kou Y, Li C, Li J, Yao M, Zhang B, Wang L, Xu H, You C, Li H, Liu S, Zhang L, Liu Y, Huang X, Tu L, Tan B, Xu Z (2024). Consistent community assembly but contingent species pool effects drive β-diversity patterns of multiple microbial groups in desert biocrust systems. Molecular Ecology.

[ref-67] Xu L, Zhang B, Wang E, Zhu B, Yao M, Li C, Li X (2021b). Soil total organic carbon/total nitrogen ratio as a key driver deterministically shapes diazotrophic community assemblages during the succession of biological soil crusts. Soil Ecology Letters.

[ref-68] Xu L, Zhu B, Li C, Yao M, Zhang B, Li X (2020). Development of biological soil crust prompts convergent succession of prokaryotic communities. Catena.

[ref-69] Xu L, Zhu B, Li C, Zhou Z, Yao M, Zhou X, Wang J, Zhang B, Li X (2021c). Increasing relative abundance of non-cyanobacterial photosynthetic organisms drives ecosystem multifunctionality during the succession of biological soil crusts. Geoderma.

[ref-70] Yao M, Rui J, Li J, Dai Y, Bai Y, Heděnec P, Wang J, Zhang S, Pei K, Liu C, Wang Y, He ZL, Frouz J, Li X (2014). Rate-specific responses of prokaryotic diversity and structure to nitrogen deposition in the *Leymus chinensis* steppe. Soil Biology and Biochemistry.

[ref-71] Yao M, Rui J, Niu H, Heděnec P, Li J, He Z, Wang J, Cao W, Li X (2017). The differentiation of soil bacterial communities along a precipitation and temperature gradient in the eastern Inner Mongolia steppe. Catena.

[ref-72] Yin Y, Li G, Xia Y, Wu M, Huang M, Zhai L, Fan X, Zhou J, Kong X, Zhang F, Riaz M (2024). How to effectively reduce sloping farmland nutrient loss and soil erosions in the Three Gorges Reservoir area. Agricultural Water Management.

[ref-73] Zhang B, Kong W, Wu N, Zhang Y (2016b). Bacterial diversity and community along the succession of biological soil crusts in the Gurbantunggut Desert, Northern China. Journal of Basic Microbiology.

[ref-74] Zhang Y, Dong S, Gao Q, Liu S, Zhou H, Ganjurjav H, Wang X (2016a). Climate change and human activities altered the diversity and composition of soil microbial community in alpine grasslands of the Qinghai-Tibetan Plateau. Science of the Total Environment.

[ref-75] Zhao J, Zhang R, Xue C, Xun W, Sun L, Xu Y, Shen Q (2014). Pyrosequencing reveals contrasting soil bacterial diversity and community structure of two main winter wheat cropping systems in China. Microbial Ecology.

[ref-76] Zheng Y, Feng Z, Wang J, Huang X, Lei L, Zhang X, Cao H, Fan D, Yao M, Han D, Li X (2021a). Wheat-root associated prokaryotic community: interplay between plant selection and location. Plant and Soil.

[ref-77] Zheng Y, Li X, Cao H, Lei L, Zhang X, Han D, Wang J, Yao M (2021b). The assembly of wheat-associated fungal community differs across growth stages. Applied Microbiology and Biotechnology.

[ref-78] Zhou X, Zhang Y, Niklas KJ (2014). Sensitivity of growth and biomass allocation patterns to increasing nitrogen: a comparison between ephemerals and annuals in the Gurbantunggut Desert, north-western China. Annals of Botany.

[ref-79] Zhu Q, Wang N, Duan B, Wang Q, Wang Y (2021). Rhizosphere bacterial and fungal communities succession patterns related to growth of poplar fine roots. Science of the Total Environment.

