# Supplementary material for: Bacterial community composition is an important predictor of surface soil fertility across different land use types: a case study in the Three Gorges Reservoir area
Source: PeerJ. 2025 Mar 27;13:e18959. doi: 10.7717/peerj.18959 (PMC11955195; doi:10.7717/peerj.18959)
Supplement: Supplemental Information 1 [file peerj-13-18959-s001.docx]

**Table S1.** Statistical summary of soil physicochemical properties in sampling plots with different land use types

| Land use | Factors ^a^ | Mean | Median | Std | Min | Max |
| --- | --- | --- | --- | --- | --- | --- |
| Sloping lands | AK (mg kg^−1^) | 43.62 | 42.40 | 0.39 | 15.45 | 88.70 |
|  | AN (mg kg^−1^) | 52.29 | 54.60 | 0.41 | 12.25 | 110.25 |
|  | AP (mg kg^−1^)^1^ | 27.90 | 10.82 | 1.44 | 1.00 | 140.29 |
|  | MC (%) | 3.96 | 3.77 | 0.14 | 3.19 | 5.12 |
|  | pH | 5.65 | 5.51 | 0.07 | 5.14 | 6.71 |
|  | SOC (mg kg^−1^) | 12.97 | 11.04 | 0.59 | 3.39 | 36.74 |
|  | TK (g kg^−1^) | 9.14 | 9.34 | 0.62 | 1.79 | 15.73 |
|  | TN (g kg^−1^) | 0.69 | 0.58 | 0.67 | 0.13 | 2.31 |
|  | TP (g kg^−1^) | 1.09 | 1.10 | 0.60 | 0.06 | 4.26 |
| Flat lands | AK (mg kg^−1^)^1^ | 76.04 | 92.85 | 38.93 | 53.92 | 126.53 |
|  | AN (mg kg^−1^)^1^ | 91.47 | 113.23 | 53.73 | 59.50 | 138.60 |
|  | AP (mg kg^−1^)^1^ | 21.01 | 35.57 | 31.47 | 4.10 | 67.23 |
|  | MC (%) | 4.03 | 4.58 | 1.17 | 3.41 | 5.02 |
|  | pH | 5.09 | 5.27 | 0.47 | 4.79 | 5.62 |
|  | SOC (mg kg^−1^)^1^ | 18.57 | 20.95 | 5.55 | 15.4 | 32.49 |
|  | TK (g kg^−1^) | 8.66 | 13.89 | 10.70 | 3.19 | 14.90 |
|  | TN (g kg^−1^) | 0.96 | 1.26 | 0.53 | 0.73 | 1.36 |
|  | TP (g kg^−1^) | 0.86 | 1.03 | 0.19 | 0.84 | 1.18 |
| Forests | AK (mg kg^−1^)^1^ | 64.49 | 52.5 | 31.88 | 28.09 | 119.52 |
|  | AN (mg kg^−1^)^1^ | 90.76 | 93.10 | 26.7 | 47.25 | 135.10 |
|  | AP (mg kg^−1^)^1^ | 3.08 | 1.26 | 3.52 | <DL ^b^ | 10.09 |
|  | MC (%) | 3.93 | 3.77 | 0.63 | 2.86 | 5.00 |
|  | pH | 5.22 | 5.28 | 0.46 | 4.17 | 5.92 |
|  | SOC (mg kg^−1^)^1^ | 21.83 | 17.86 | 9.49 | 9.23 | 48.38 |
|  | TK (g kg^−1^) | 9.98 | 9.39 | 6.77 | 2.60 | 20.44 |
|  | TN (g kg^−1^) | 0.92 | 0.80 | 0.60 | 0.20 | 2.56 |
|  | TP (g kg^−1^) | 0.60 | 0.63 | 0.26 | 0.14 | 1.04 |

^a^ More information about the abbreviations of the factors is given in Section 2.2 of this article.

^b^ DL: detection limit.
