# Supplementary material for: Bacterial community composition is an important predictor of surface soil fertility across different land use types: a case study in the Three Gorges Reservoir area
Source: PeerJ. 2025 Mar 27;13:e18959. doi: 10.7717/peerj.18959 (PMC11955195; doi:10.7717/peerj.18959)
Supplement: Supplemental Information 2 [file peerj-13-18959-s002.docx]

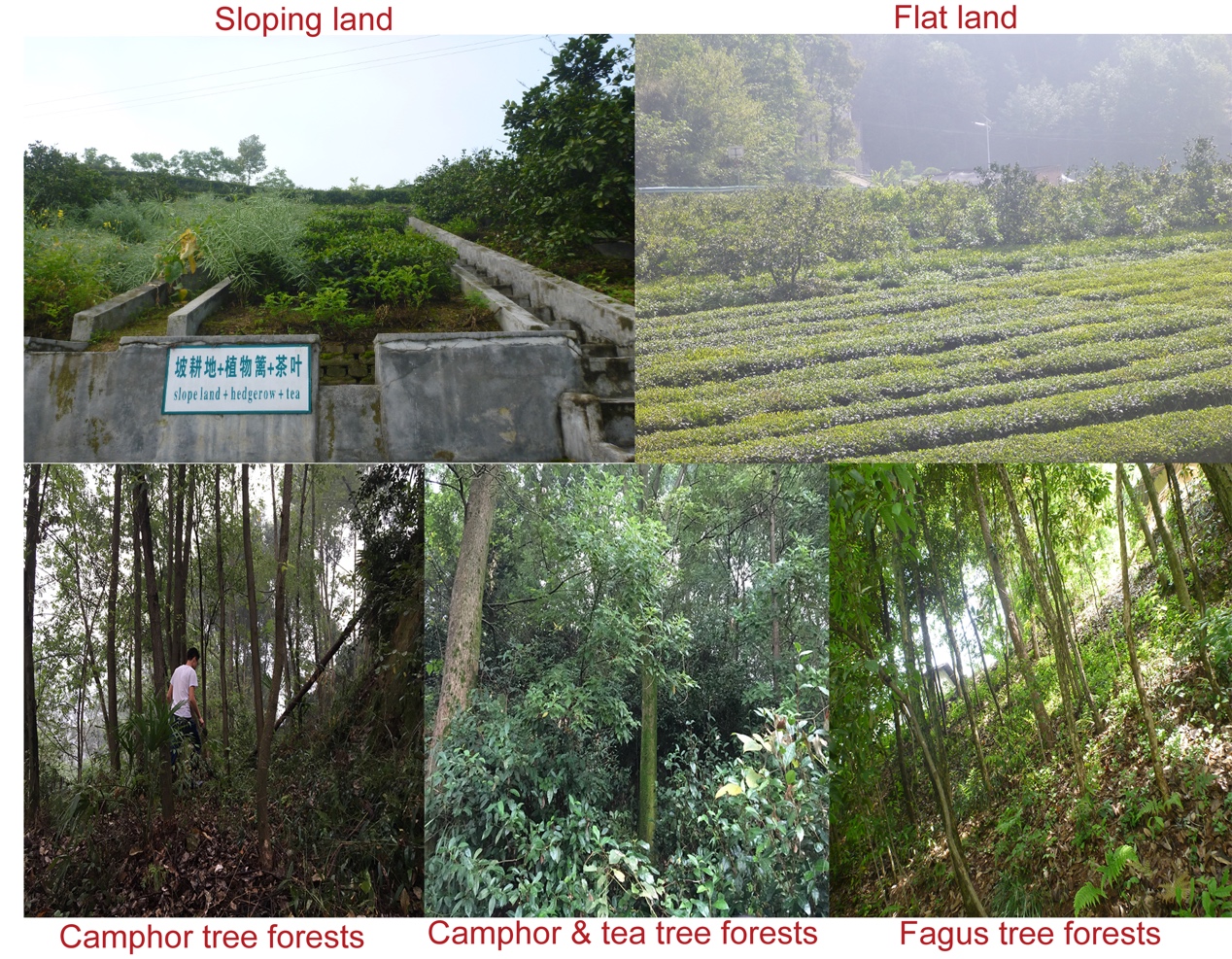


**Figure S1.** Photographs demonstrating the natural condition of each land use type sampled. The images were taken by Lin Xu and Zhongsai Tian during sampling.

**
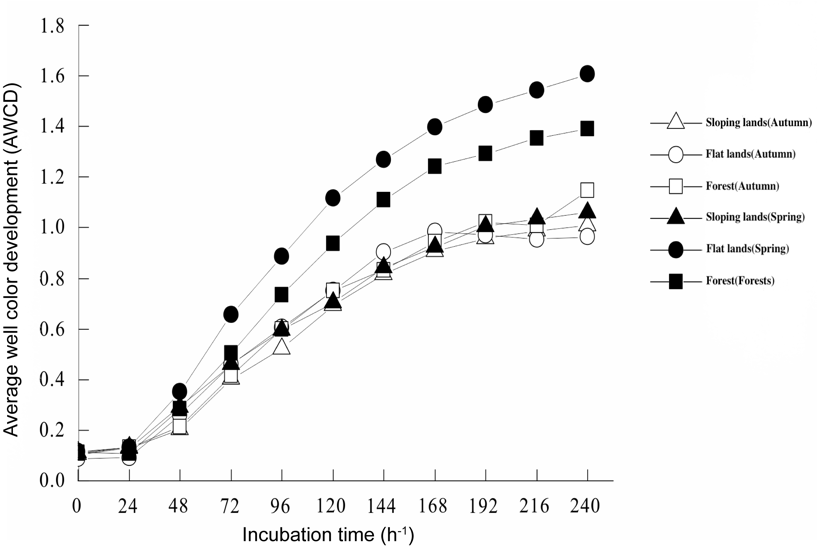
**

**Figure S2.** Average well color development dynamics (AWCD) with the incubation of soil microorganisms at different times.

**Figure S3.** Correlations between soil fertility index and each individual soil fertility. The number in each block indicates significance of the Spearman’s correlation *rho*: ^*^, *P* < 0.05; ^**^, *P* < 0.01; ^***^, *P* < 0.001.


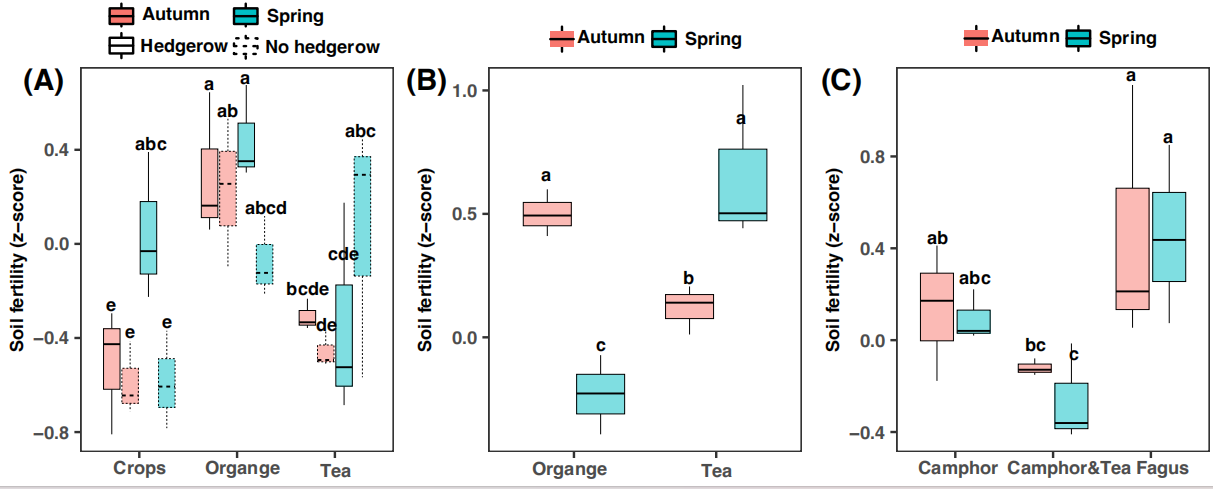


**Figure S4.** Boxplots of soil fertility varying temporally among plots dominated by different plants and in different land use types (A:sloping land, B: flat land,C:forest). Each boxplot includes three samples (n = 3). Difference lower-case letters indicate significant differences determined by the Kruskal-Wallis test at a significance level of *P* < 0.05.

**
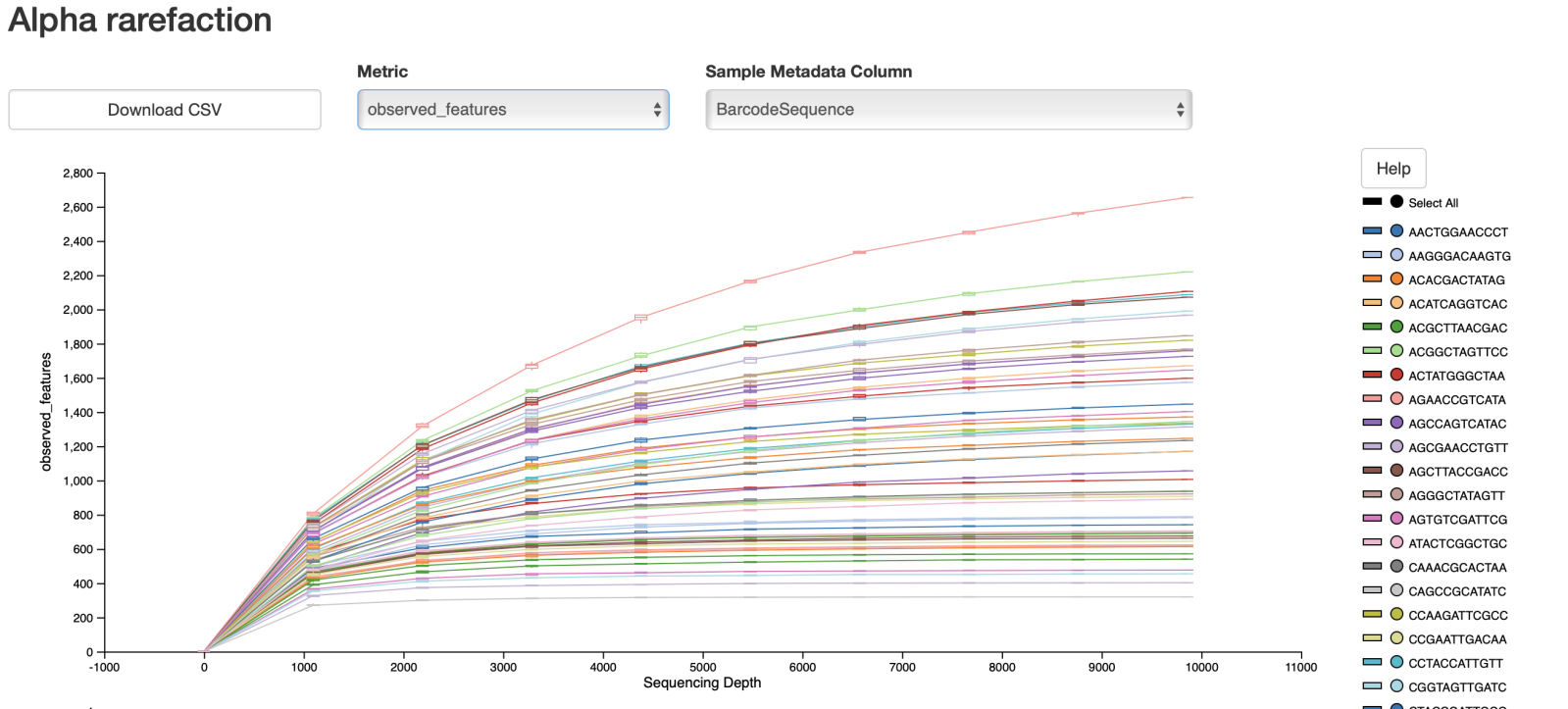
**

**Figure S5.** Rarefaction curves showing the relationships between sequencing depth and observed ASVs numbers of soil bacterial communities.


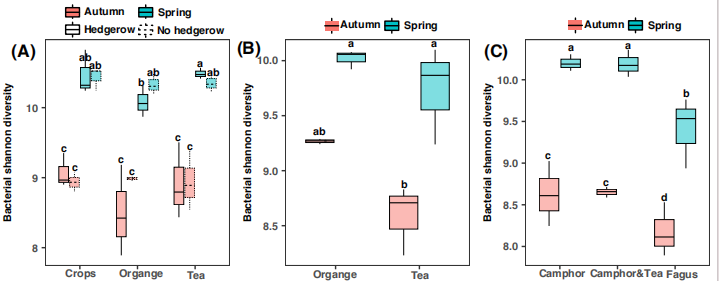


**Figure S6.** Boxplots of bacterial Shannon diversity varying temporally among plots dominated by different plants and in different land use types (A:sloping land, B: flat land,C:forest). Each boxplot includes three samples (n = 3). Difference lower-case letters indicate significant differences determined by the Kruskal-Wallis test at a significance level of *P* < 0.05.


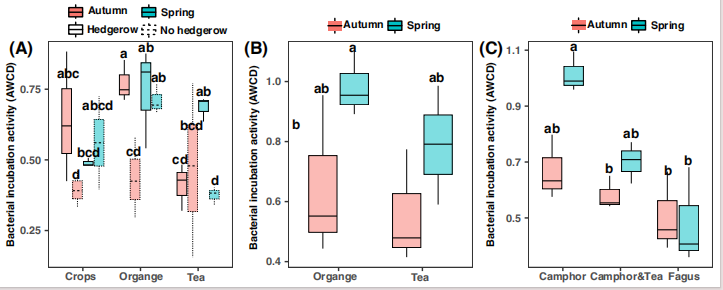


**Figure S7.** Boxplots of microbial metabolic activity (average well color development, AWCD) varying temporally among plots dominated by different plants and in different land use types (A:sloping land, B: flat land,C:forest). Each boxplot includes three samples (n = 3). Difference lower-case letters indicate significant differences determined by the Kruskal-Wallis test at a significance level of *P* < 0.05.

**Figure S8.** Correlations between soil fertility index and relative abundances of key bacterial phyla identified by the random forest modelling. The number in each block indicates significance of the Spearman’s correlation test: ^*^, *P* < 0.05; ^**^, *P* < 0.01; ^***^, *P* < 0.001.
